# Champion Device Architectures for Low-Cost and Stable Single-Junction Perovskite Solar Cells

**DOI:** 10.1021/acsmaterialslett.3c00337

**Published:** 2023-08-08

**Authors:** Thomas Baumeler, Amina A. Saleh, Tajamul A. Wani, Siming Huang, Xiaohan Jia, Xinyu Bai, Mojtaba Abdi-Jalebi, Neha Arora, Michael Grätzel, M. Ibrahim Dar

**Affiliations:** † Laboratory of Photonics and Interfaces, Institute of Chemical Sciences and Engineering, École Polytechnique Fédérale de Lausanne, Lausanne 1015, Switzerland; ‡ Department of Chemistry, School of Science and Engineering, The American University in Cairo, AUC Avenue, P.O. Box 74, New Cairo, 11835, Cairo Egypt; § Department of Materials Science and Engineering, Indian Institute of Technology Delhi, New Delhi, 110016, India; ∥ Institute for Materials Discovery, University College London, Malet Place, London, WC1E 7JE, United Kingdom; ⊥ Cavendish Laboratory, Department of Physics, University of Cambridge, Cambridge CB3 0HE, United Kingdom; ¶ Department of Chemistry, University College London, London, WC1H 0AJ, United Kingdom

## Abstract

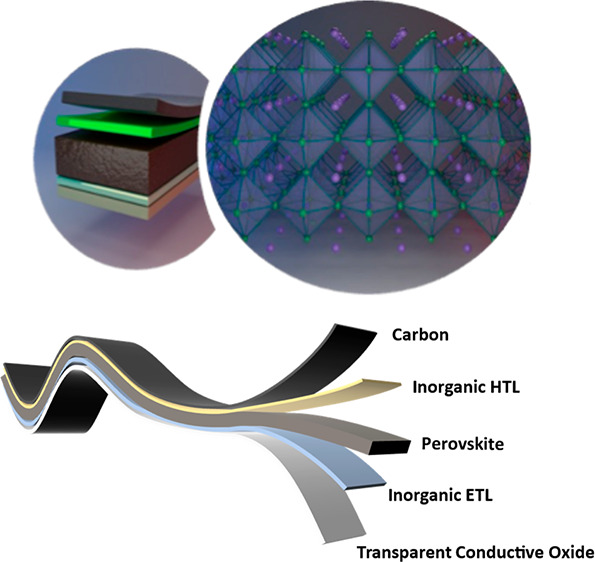

High power conversion efficiencies (PCE), low energy payback time (EPBT), and low manufacturing costs render perovskite solar cells (PSCs) competitive; however, a relatively low operational stability impedes their large-scale deployment. In addition, state-of-the-art PSCs are made of expensive materials, including the organic hole transport materials (HTMs) and the noble metals used as the charge collection electrode, which induce degradation in PSCs. Thus, developing inexpensive alternatives is crucial to fostering the transition from academic research to industrial development. Combining a carbon-based electrode with an inorganic HTM has shown the highest potential and should replace noble metals and organic HTMs. In this review, we illustrate the incorporation of a carbon layer as a back contact instead of noble metals and inorganic HTMs instead of organic ones as two cornerstones for achieving optimal stability and economic viability for PSCs. We discuss the primary considerations for the selection of the absorbing layer as well as the electron-transporting layer to be compatible with the champion designs and ultimate architecture for single-junction PSCs. More studies regarding the long-term stability are still required. Using the recommended device architecture presented in this work would pave the way toward constructing low-cost and stable PSCs.

The unprecedented increase in the power conversion efficiency from an initial value of 3.81% in 2009^1^ to over 26%^2,3^ establishes perovskite solar cells (PSCs) as one of the most promising PV technologies which exhibits the potential to compete and intergrade in tandem structures with silicon PV cells.^4^ Although the metal halide perovskite (MHP) semiconductors offer reasonable flexibility, tunable properties, lightweight and semitransparency, the poor stability and high costs of PSCs are still impeding their commercialization and large-scale deployment.^5,6^ The use of evaporated noble metals (such as gold (Au)) as back electrodes in the PSCs yielding record power conversion efficiency (PCE) values represents one major contributor to this impending bottleneck, due to their prohibitive cost and highly energy-consuming deposition methods.^6^ Furthermore, metal electrodes participate in forming gold/silver halide species,^7−9^ and such phenomena hinder the long-term stability of perovskite PV devices.^10^ In this regard, carbon electrodes represent a very interesting alternative. Carbon electrodes not only are much cheaper than their noble metal counterparts but also offer more desirable features such as suitable energy levels, simple manufacturing processes, and flexibility.^11−14^

In addition to the costs and stability issues of precious metals, the hole transport material (HTM) employed in device architecture yielding remarkable efficiency values present another set of major economical and sustainability challenges.^15a^ First, organic HTM usually has the lowest thermal stability of all layers in the PSC, when to-date’s benchmark organic HTM 2,2′,7,7′- tetrakis(N,N-dipmethoxyphenylamine)-9,90-spiro-bifluorene (spiro-OMeTAD) is employed, mostly due to the hygroscopic and mobile nature of the dopants (such as Li^+^) required to enhance the hole mobility and conductivity.^15b^ Moreover, the commercially available high-purity spiro-OMeTAD is almost ten times more expensive than gold (∼400 $/gram), preventing any penetration of such PSCs to the PV market.^16^ The use of inorganic hole conductors as a replacement for their organic counterparts is promising, given that inorganic HTMs do not require complicated, low-yield multiple-step synthesis, making them much cheaper materials (affordable for only ∼1 $/gram). Furthermore, intrinsically p-type inorganic materials exhibit excellent mobility without the need for doping and thus offer superior chemical and photothermal stability compared to their organic counterparts.^17a^

This review focuses on the recent development of potentially the best single-junction PSC architectures that would lead to the highest possible efficiency, the lowest cost available, and optimal stability. Designing architectures where precious metal and organic HTM are replaced by carbon-based back contact and inorganic HTM, respectively, seems to be the most viable route that could yield stable devices in a low-cost framework with a satisfactory PCE and operational stability. Further improvements in the electron transporting layer (ETL) and the perovskite layer are also considered to opt for champion single-junction device architectures. Finally, we believe that semitransparency is a complementary property in the PSCs that needs to be explored if PSC-based tandem devices are alternatively investigated, pushing the commercialization, viability, and efficiency of solar cells to the ultimate level.

## Device Architecture and Working Principle

**Figure 1:**
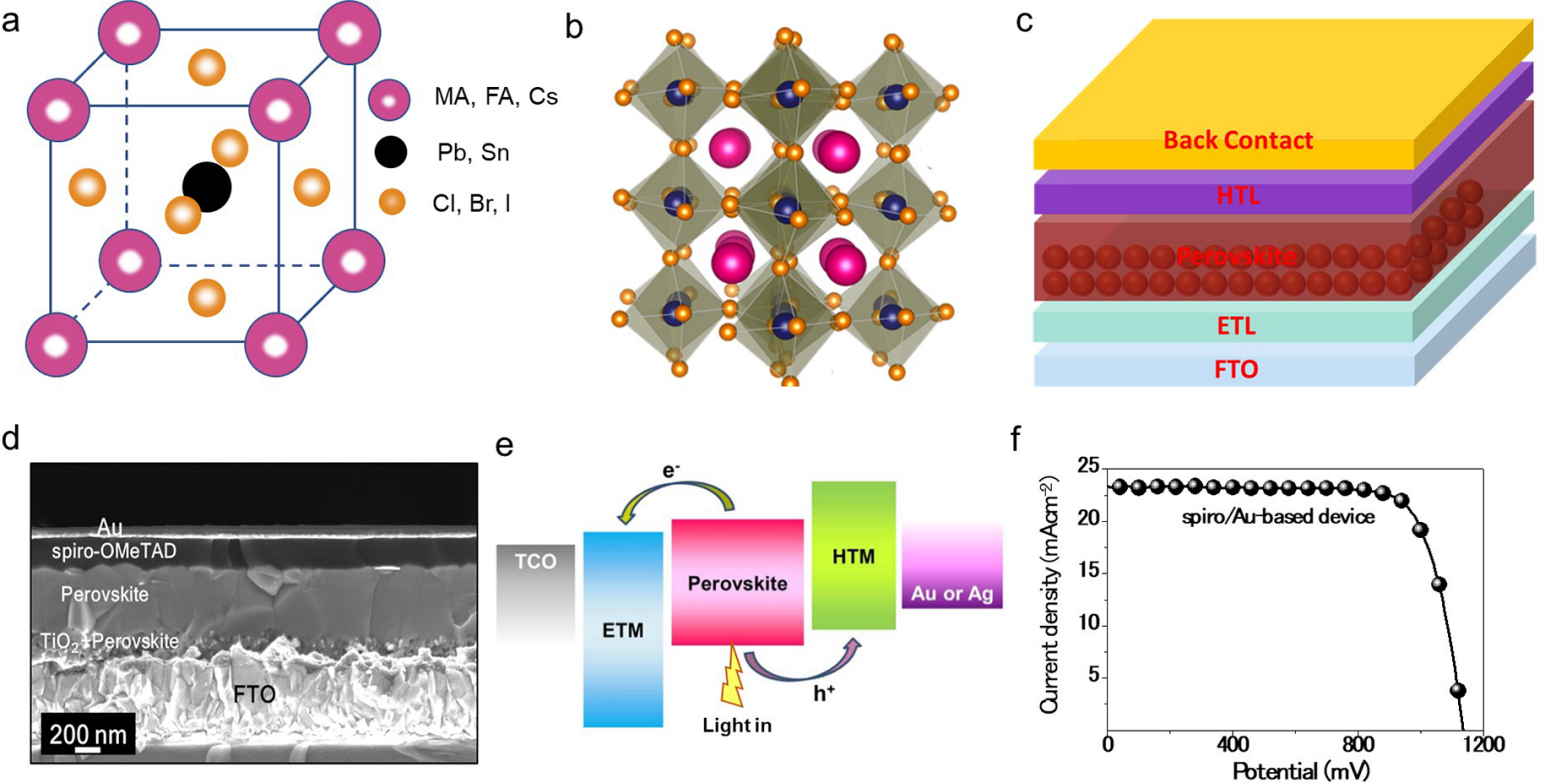
Structure of light absorber and solar cell with energy levels and current–voltage characteristic. (a) Schematic diagram of the unit cell of ABX_3_ perovskite crystal structure. (b) 3D schematic diagram of ABX_3_ perovskite crystal structure. (b) Reproduced with permission from ref (17c). License CC BY 4.0. https://creativecommons.org/licenses/by/4.0/. Copyright 2023 The Authors. Published by American Chemical Society. (c) Schematic stack structure of n–i–p perovskite solar cells. (d) Cross-sectional SEM image, (e) energy level diagram, and (f) *J*–*V* curve of a typical perovskite solar cell. (d, f) Reproduced with permission from ref (18a). Copyright 2017 *Science*.

Metal halide perovskite materials have a general crystal structure of ABX_3_, where A can be an organic (e.g., CH_3_NH_3_^+^) or inorganic (e.g., Cs^+^) cation, B is an inorganic cation (Pb^2+^ or Sn^2+^), and X is a halide anion (e.g., Cl^–^, Br^–^, I^–^) (Figure 1a, 1b). A and B cations can coordinate with 12 and 6 X anions, resulting in cuboctahedral and octahedral geometries, respectively.^17^ In a conventional n–i–p architecture (Figure 1c), an ETL exhibiting a wide bandgap is deposited over a transparent conducting oxide (TCO), so that maximum solar light can first pass through it.^17e^ The perovskite material is then deposited onto the ETL (which may entail an optional mesoporous scaffold layer) and capped by an HTM followed by a back contact electrode (typically Au or Ag) as shown in Figure 1c and 1d.^18a^ While designing the selective contacts, i.e., ETL and HTM, the energy levels corresponding to the valence and conduction bands should be well aligned to facilitate charge transport across the layers and fully assembled devices.

Figure 1e schematically illustrates the energy level diagram of this configuration and shows how electrons and holes get generated and collected under illumination.^18b^ First, the perovskite absorbing layer is photoexcited, resulting in the excitation of electrons from the valence band (VB) to the conduction band (CB). Excitons or free charge carriers can be formed after the photoexcitation. Charge separation then occurs: the photogenerated electrons are injected into the CB of the ETL, and the photogenerated holes are injected into the VB or highest occupied molecular (HOMO) orbital level of HTM.^19^ These injection processes are possible only because of energy matching: the conduction band of the ETL is lower than that of the perovskite, and the HOMO level or the VB of the HTM is higher than the valence band of perovskite.^15a^

The current–voltage response of a typical PSC is represented in Figure 1f. To achieve devices with high performance, the kinetics of the previously mentioned injection processes must be much faster than those of all the other competing recombination processes. That is, the created carriers must reach the appropriate interfaces before they recombine, or else the collection efficiency would drop.^18a^ Therefore, it is crucial to choose the proper layer to optimize the PSC’s performance (Figure 1f).

## Inorganic Hole Conductors

Hole conductors play a critical role in obtaining efficient solar cells, as they lower the transporting barrier, extract holes from perovskites, block the electron transport between the perovskite and the electrode, and minimize the charge carrier recombination (Figure 1e).^18b^ The use of inorganic HTMs as a replacement for their organic counterparts is driven by both economic and sustainable logics: inorganic materials are much cheaper, thermally and chemically more stable, and solution processable. They also offer other suitable properties, such as wide bandgap and high optical transmittance.^20^ A broad array of suitable inorganic HTMs include copper-based materials, nickel oxide (NiO_x_), MoS_2_, and molybdenum oxides, etc.^17a^

### Current State of Art

**Figure 2:**
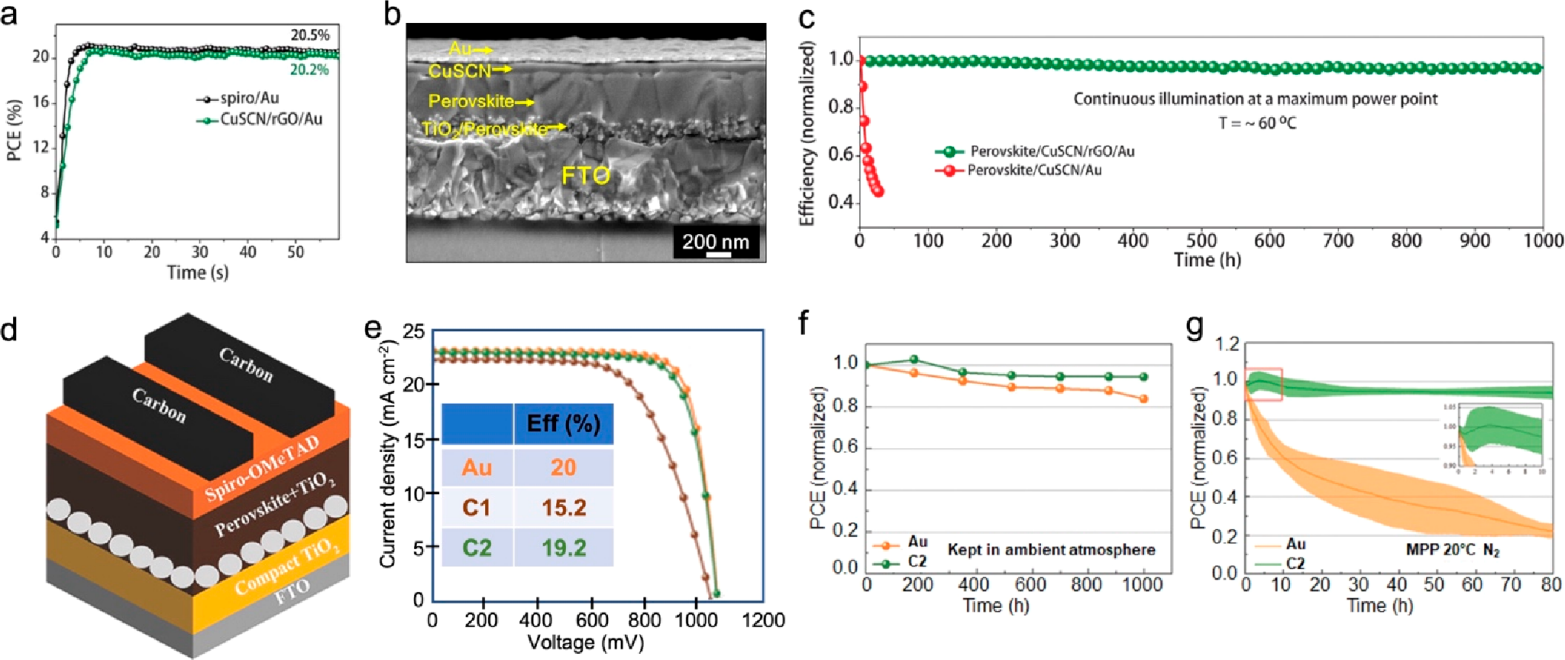
Efficiency and stability of inorganic hole conductor-based solar cell: (a) maximum power point tracking for 60 s, yielding stabilized efficiencies of 20.5% and 20.2%, respectively, for spiro-OMeTAD–based and CuSCN-based devices; (b) cross-sectional of the CuSCN PSC device; and (c) operational stability of an unencapsulated CuSCN-based device with and without a thin layer of rGO (as a spacer layer between CuSCN and gold layers). (a–c) Reproduced with permission from ref (18a). Copyright 2017 *Science*. (d) Schematic diagram of mesoporous C-PSCs. (e) *JV* curves of PSCs with Au, C1 (carbon electrode formed by heating a wet carbon film), and C2 (carbon electrode formed by solvent-exchange of a wet carbon film) as electrodes, (f) shelf stability kept in the ambient atmosphere without any encapsulation, and (g) operational stability under constant illumination of the same C2-and Au-based PSC devices. (d–g) Reproduced with permission from ref (26). Copyright 2018 Wiley.

In terms of performance, inorganic HTMs have already reached reasonably high PCE values. CuSCN, NiO_*x*_, MoS_2_, and Co_3_O_4_–SrCO_3_ were all employed as HTMs in PSCs demonstrating >20% PCE, with a reported PCE of 20.4% for CuSCN (Figure 2a),^18a^ 20.6% using NiO_x,_^21^ 20.4% for MoS_2_,^22^ and 21.84% for Co_3_O_4_–SrCO_3,_^23^ exhibiting to date the highest potential for PSCs employing an inorganic HTM. Interestingly, the PSC architectures and the HTM deposition methods that yield the best PCE differ for each respective inorganic HTM. For CuSCN, the highest efficiency is reached when CuSCN is employed in a mesoscopic n–i–p architecture and deposited by the dynamic spin-coating method (Figure 2b).^18a^ Regarding nickel oxide-based planar p–i–n architecture, PCEs overpassing the 20% are reached using both spin-coating and spray deposition methods,^21,24^ whereas MoS_2_ overpass the 20% PCE in a mesoscopic n–i–p architecture via spray coating deposition.^22,25^ Co_3_O_4_–SrCO_3_ HTM represents a self-organized percolative architecture composed of narrow bandgap oxide, Co_3_O_4_, and wide bandgap oxysalt, SrCO_3_ used in a p–i–n architecture. Noticeably, the reported 20.4% efficiency reached by Arora et al. with CuSCN and by Kohnehpoushi et al. using MoS_2_ was achieved using a pristine, undoped HTM layer, whereas highly efficient PSCs based on NiO_*x*_ require the doping of the nickel oxide layer with Cs, Cu, and/or Li.^21,24^

### Stability

The most crucial technologic parameter required to deploy PSCs is to effectively produce stable perovskite devices, ensuring a long lifetime of the photovoltaic panels. In that regard, the organic HTM presents a major challenge as it usually has the lowest thermal stability of all layers in the PSC when the benchmark organic spiro-OMeTAD is employed, primarily because of the hygroscopic and mobile nature of the doping ions needed to improve transport properties.^15^ The use of inorganic layers has led to a drastic improvement in terms of stability over organic HTMs, as inorganic compounds are, in general, much more stable from both photothermal and photochemical perspectives than organic ones. As proof of that, much-improved stability is observed for PSCs fabricated using CuSCN, NiO_*x*_, or MoS_2_ when compared to those using an organic spiro-OMeTAD- or PEDOT:PSS-based HTM.^25,27−29^ Moreover, Arora et al. reported PSCs employing copper(I) thiocyanate as an HTM with a stabilized efficiency retaining more than 95% of their initial 20.4% PCE, after aging at maximum power point (MPP) for 1000 h under full sun intensity at 60 °C,^18a^ setting another example of the stability of inorganic HTMs (Figure 2c).

### Costs and Processability

Overcoming the low stability and high costs of organic HTMs, inorganic HTMs represent a major potential return on investment for the PSCs industry. Generally, inorganic chemicals suitable for hole transport purposes are relatively economical (≈ 1 $/gram), compared to their benchmark organic counterpart, with a hundred- to 1000-fold diminution on the costs per gram of material (≈ 400 $/gram for spiro-OMeTAD for instance). Such a drastic diminution of the costs related to the HTM would certainly benefit the PSC industry. However, to truly assess the extent to which this cost reduction is significant, the proportion of the cost related to the HTM must be rationalized with the overall final production costs of a PSC. Interestingly, Li et al. calculated the cost for a PSC module based on 1 cm^2^ 19% efficient planar solar cells using a large area screen printing method to deposit the different layers (SnO_2_ electron transport layer, MAPbI_3_ perovskite, NiO_*x*_ HTM, and copper electrodes). The replacement of the nickel oxide by spiro-OMeTAD induces a significant jump of 348% in the module production costs and a 166% increase in the levelized cost of electricity (LCOE, represents the unit cost (per kilowatt hour) of electricity over the lifetime of a certain generating entity).^30^ Thus, the change to cheaper hole transport layers is of prime importance for the PV industry, indicating that usage of expensive material is probably one of the major reasons that prevent any market penetration of PSCs.

Regarding processability, inorganic charge transport layers offer an extensive array of different deposition methods suitable for industrial standards, such as atomic layer deposition (ALD), pulsed-laser deposition (PLD), electrodeposition, etc.^31−33^ It is crucial, however, to check whether such methods can yield efficient photovoltaic devices. From this perspective, the three main candidates of inorganic HTMs (CuSCN, NiO_*x*_, and MoS_2_) show the potential to deliver highly efficient (>20% PCE) PSCs. In addition, inorganic HTMs are more suitable toward large-scale industrial development, as spray deposition can readily be used to deposit high-quality films of large areas and has even been shown to be applied to PSCs.^34^

## Carbon Electrode-Based PSCs

The back electrode, the uppermost layer in PSCs is most exposed to the environment. It should thus be robust enough to minimize moisture penetration into the perovskite layer. Typically, Au or Ag is employed as the back contact, but these precious metals are expensive and require energy-intensive deposition methods. Besides, they are unstable and can cause severe, irreversible degradation to the device: Ag reacts with the halide (diffused from the perovskite layer) to form silver halide, whereas Au diffuses across the HTM into the perovskite and causes perovskite decomposition. In contrast, carbon electrodes are cheap, resistant to moisture, and flexible and can be processed via simple deposition methods.

The back contact in PSCs needs to show superior electrical conductivity^35−37^ and the energy levels of the back electrode also need to match those of the perovskite or the HTM to extract and collect charges efficiently.^38^ In that regard, carbon is particularly promising since various work functions (Fermi levels) can be achieved to optimize charge extraction by varying the carbon species. For instance, carbon black shows a work function of 4.6–5.0 eV,^39,40^ carbon nanotubes (CNTs) exhibit 4.7–5.0 eV,^41,42^ graphite 4.4–4.7 eV,^43^ graphene oxide 4.9 eV, and graphene 4.2–4.6 eV.^44^

To facilitate the charge separation process as well as the extraction, the Fermi level of the carbon electrode must be close to that of the perovskite material.^45^ With that respect, carbon offers tunability to adapt the Fermi level of the electrode to that of the perovskite/HTM. For example, Li et al. prepared a single-walled carbon nanotube (SWCNT)/graphite/carbon black (1:4:1 in mass ratio) composite in which the SWCNT acted as the hole transporting layer with the charge extraction taking place only at the perovskite/carbon interface and the conductivity being determined by the bulk carbon. The work function was tuned via the amount of SWCNT. As a result, the charge collection was increased compared to that without the SWCNT additive, resulting in carbon-based PSCs (C-PSCs) exhibiting higher PCE.^46^ In some other cases, it is still a matter of debate in the scientific community if the carbon layer behaves simply like an ohmic contact or has HTM properties.

### PCE Evolution over the Years

Achieving high PCE from PSCs employing carbon electrodes is a significant challenge: effectively, C-PSCs yield lower PCE than those based on noble metal electrodes.^47^ This phenomenon is especially true when no HTM is used as the perovskite absorber comes in direct contact with the carbon electrode: poor contact between the perovskite and the subsequently deposited carbon layers negatively affects the hole transport process, preventing efficient charge extraction and promoting nonradiative recombination, leading to poor efficiency devices.^48−50^ In 2013, for the first time the use of carbon/graphite electrodes in PSCs was documented and 6.64% PCE was obtained.^51^ This work was followed by Ma’s group report on the fabrication of low-cost TiO_2_/CH_3_NH_3_PbI_3_ (MAPbI_3_)/carbon photovoltaic devices where the carbon electrode was formed through a low-temperature process (70 °C), yielding 9.0% PCE.^52^ Later the same year, Yang et al. reported 10.2% efficient C-PSCs via the use of a mesoscopic carbon layer and flexible graphite paper to form an all-carbon electrode^35^ and they pushed further their work on flexible carbon electrodes by hot pressing a free-standing thermoplastic carbon film onto the perovskite layer, delivering 13.5% PCE devices.^53^

In 2016, Li et al. came up with 14.7% efficient C-PSCs by doping the graphite/carbon black with SWCNTs, enhancing the charge collection and thus, the PCE,^54^ while Zhang et al. reported 16.1% PCE by applying carbon on top of a Copper phathalocyanine (CuPc) nanorods HTM.^47^ In 2017, Mamun et al. reported 16.2% efficient PSCs by combining carbon with PCBM to form a very flat carbon layer using an e-beam irradiation method.^55^ Noticeably, the carbon/PCBM layer demonstrated a better interface defect passivation effect and higher conductivity than that of pure PCBM and C_60_/PCBM layers. In the same year, HTM-free C-PSCs jumped to 15.3% PCE employing boron-doped multiwalled carbon nanotubes (MWCNTs) as back contact.^56^ In the later work, the replacement of Au electrode by low-temperature-processed MWCNTs improved the PCE from 12.81% to 15.6% and drastically reduced the hysteresis.^57^

The main issue, which prevented reaching higher efficiency, when using carbon paste electrodes came from the fact that commercial carbon pastes contain solvents that can create bulges and pinholes in the material during evaporation.^58^ To overcome this, Zhang et al. came up with a solution by fabricating a self-adhesive carbon film processed at room temperature by solvent exchange method (Figure 2d).^26^ The paste was doctor-bladed on glass, soaked in ethanol, and dried. It was then removed from the glass and pressed onto the HTM layer, leading to an impressive 19.2% efficient C-PSCs, very close to the value for the same perovskite composition by using a gold electrode (Figure 2e). The key feature here is that the self-adhesive carbon electrode can readily form an excellent, defect-free interface contact with the HTM. Peng et al. used a similar approach to apply carbon electrodes to PSCs and achieved 19.36% PCE.^59^ In the field of HTM-free C-PSCs, the record PCE was reported by Chen et al. In their work, mesoporous carbon electrodes were used in fully printable PSCs to achieve 17.47% PCE in a FAPbI_3_-based architecture and 16.24% for MAPbI_3_-based devices.^60^ A fluorinated 2D wide-band gap perovskite (F_5_PEA_2_PbI_4_) was used as an electron blocking layer at the 3D perovskite/carbon electrode interface, allowing for improved photovoltage (*V*_OC_) and reduced halide migration. This is a remarkable result, as flexible devices enable the tunability to adapt PSCs for a wide array of different uses. Furthermore, the excellent potential of low-cost carbon-based device design strategy for large-scale deployment was demonstrated.^61^

### Stability

It is well-known that the constituent ions of metal halide perovskite materials are mobile in the solid state and can participate in reduction/oxidation reactions.^62^ When these halide ions move to the electrode, the gold/silver electrode undergoes electrochemical oxidation, and mobile gold/silver ions are created, forming gold/silver halides, leading to the deterioration of the perovskite/electrode or HTM/electrode interface.^6−9^ As a result, the PSC efficiency drops dramatically fast. From that perspective, moving from noble metals to carbon electrodes represents a major step forward toward the fabrication of highly stable PSCs. Moreover, because of its hydrophobicity, the use of carbon electrodes provides the very desirable feature of moisture protection for the PSCs, which is another key feature toward achieving long-lasting PSCs.^58,63,64^Figure 2f–g illustrates the improvement in the stability of PSCs when the Au is replaced by carbon, as reported by Zhang et al.^26^ As can be seen, shelf stability is slightly improved upon the application of carbon contacts, and the operational stability (measured at MPP conditions, at 20 °C in a N_2_ atmosphere) is drastically improved. However, the length of the measurement is only 80 h.

**Figure 3:**
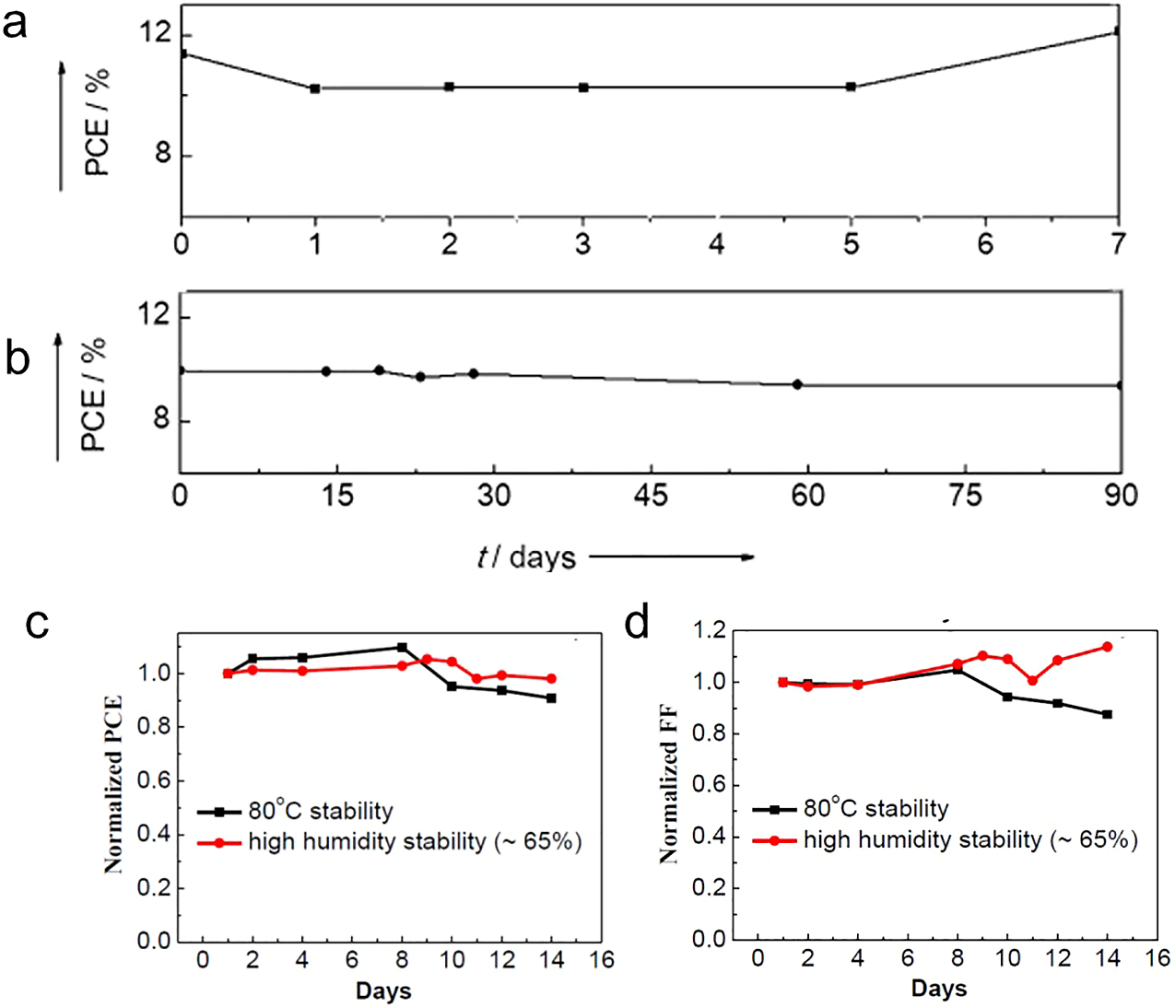
(a) Time evolution of the encapsulated PSC solar cell metrics during outdoor aging in Jeddah, Saudi Arabia and (b) indoor heat stress test of a triple-layer PSCs. (a, b) Reproduced with permission from ref (65). Copyright 2015 Wiley. (c) Normalized PCE and (d) FF of two TiO_2_/Al_2_O_3_-BMWNTs-PSCs as a function of storage time at 80 °C and at a high humidity of ≈65%. (c, d) Reproduced with permission from ref (56). Copyright 2017 American Chemical Society.

Harsher conditions were even tested outdoors to prove the stability of carbon-based devices. In 2015, Li et al. reported on the stability of hole-conductor-free MAPbI_3_ C-PSCs based on a triple-layer architecture employing carbon as a back contact and delivering 10–12% PCE.^65^ They performed outdoor tests in the hot desert climate, and long-term indoor light soaking and heat exposure for 3 months at 80–85 °C. Interestingly, encapsulated PSCs tested outdoors in Jeddah, Saudi Arabia for 1 week (September 7–14, 2014) demonstrated excellent stability, as their PV parameters remained remarkably stable over the 7 day period, and the final PCE values were even slightly above the initial ones (Figure 3a). Heat-stress measurements were also carried out indoors, as PSCs were encapsulated and kept for 3 months in a normal oven filled with ambient air at 80–85 °C (Figure 3b). They were removed at several intervals from the oven and cooled overnight to equilibrate at ambient temperature before recording the performance metrics. Measurements employed simulated one solar AM 1.5 light at room temperature. In that case, the triple-layer devices demonstrated stable PV parameters (within a few percent) as well. Finally, they measured the long-term photostability of their PSCs by performing indoor light-soaking tests under continuous illumination with a white light-emitting diode (LED) array, emitting visible light at an intensity of 100 mW/cm^2^ for 1056 h. The photovoltaic metrics were recorded every 6 h by computer-controlled measurements of the *JV* curve. Again, these parameters remained remarkably stable, with less than 1% relative loss of PCE over the 1056 h, showing no evidence of any significant performance degradation under these conditions.

The steady-state stability of a 70 cm^2^ carbon-based HTM-free perovskite module delivering >10% PCE was investigated by carrying out 6 steady-state current measurements at MPPV for 72 h over a period of 2000 h (84 days) under ambient conditions (65–70% relative humidity (RH) and 25–30 °C).^66^ Impressively, the module showed high stability, as they reported less than a 5% (relative) drop in efficiency and showed that the module efficiency increased after 72 h of testing. The critical role in such stability was attributed to the hydrophobic top carbon layer, which prevents moisture-related degradation of the perovskite crystals. Finally, they also showed an excellent reproducibility of the modules, with 18 devices having a PCE standard deviation of only 0.65%, which is another key factor toward industrialization.

Zheng et al. came up with >15% PCE HTM-free PSCs using a MAPbI_3_ perovskite and boron-doped multiwall carbon nanotubes (B-MWNT) to form the electrode.^56,67^ At the time, they investigated the shelf stability of their devices by storing them under dry air, under heating stress (80 °C) and under high humidity (≈65% RH at 25 °C). The devices remained stable in dry air (98% of initial PCE retained after 80 days of storage) and lost 15% and 7% of the initial PCE at 80 °C and 65% RH, respectively (Figure 3c–d).^56^ The stability was attributed to the hydrophobic character of the CNTs and further extended to the formation of compact interlinked MWNTs’ network films.

**Figure 4:**
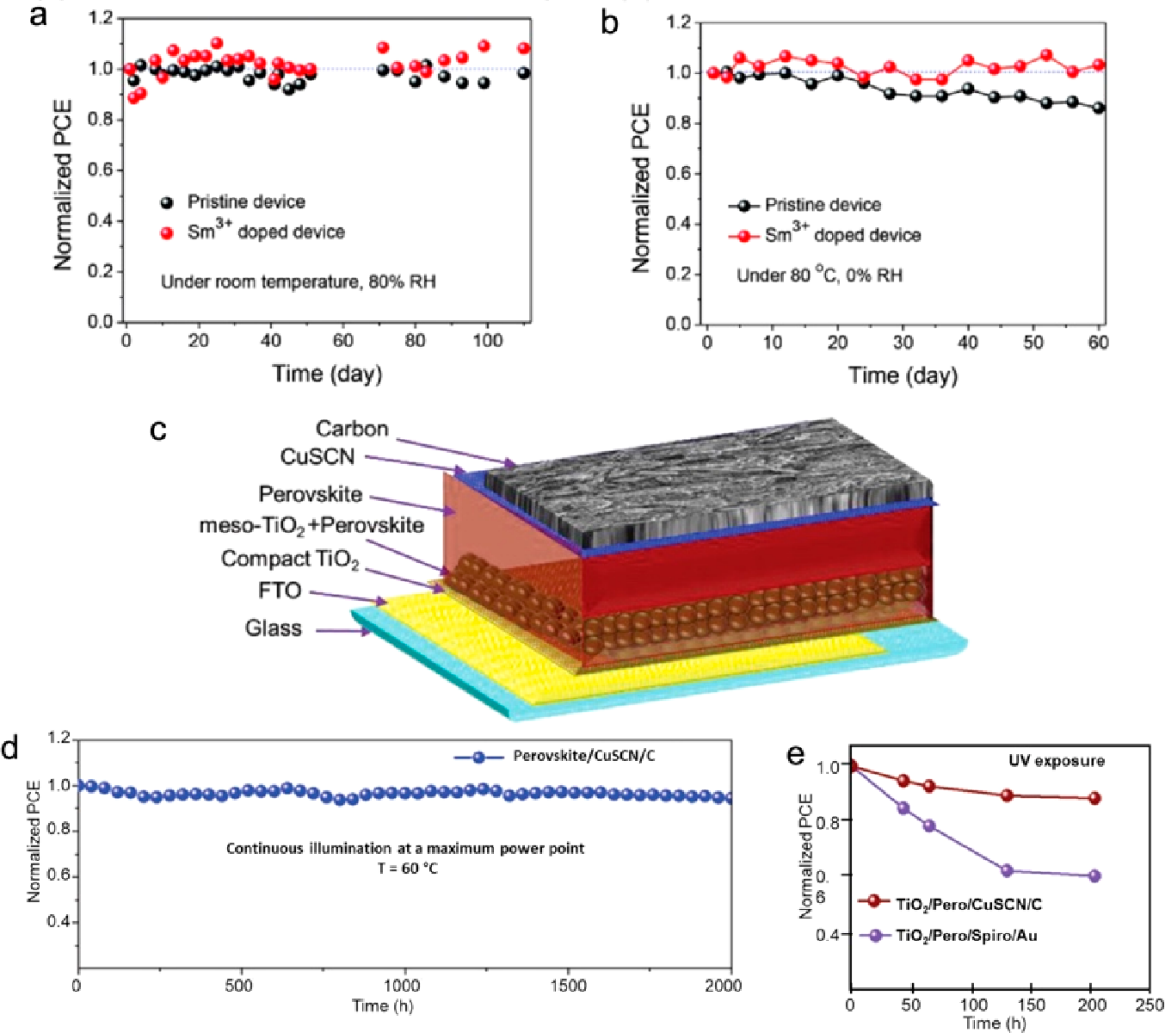
Long-term stability of the pristine and Sm^3+^ doped devices without encapsulation under (a) 25 °C and 80% RH and (b) 80 °C and 0% RH. (a, b) Reproduced with permission from ref (68). Copyright 2018 Wiley. (c) Schematic diagram of PSC with the device architecture FTO/compact-TiO_2_/meso-TiO_2_/Perovskite/CuSCN/C and (d) operational stability of the same latter device, as reported by Arora et al. for 2000h at MPP conditions (AM1.5), and (e) UV stability comparison of Au- and carbon-based devices. (c–e) Reproduced with permission from ref (27). Copyright 2019 Wiley.

A fully inorganic HTM-free PSCs architecture based on lanthanide-doped CsPbBr_3_ and carbon black as CE was reported.^68^ They demonstrated a PCE of 10.14% with an ultrahigh *V*_OC_ of 1.594 V for an FTO/c-TiO_2_/m-TiO_2_/CsPb_0.97_Sm_0.03_Br_3_/carbon PSC under one sun illumination. As shown in Figure 4, these devices showed excellent long-term stability even at 80% RH at 25 °C (Figure 4a) or 80 °C (Figure 4b). In the case of 25 °C and 80% RH, the Sm^3+^-doped PSCs exhibited a 10% (relative) increase in the PCE after 110 days, and the PCE remained stable for 60 days at 80 °C and 0% RH, whereas undoped PSCs showed significant degradation (90% and 80% of the initial PCE, respectively). In addition to the hydrophobic effect of the carbon CE, the doping with Sm^3+^ ions increased the stability by lattice contraction, similarly as reported by Zou et al. upon the doping of cesium lead halide perovskites using Mn^2+^ ions.^69^

While the previous examples of works showed highly stable HTM-free C-PSCs, other inorganic HTM C-PSCs showed equally impressive stable devices, along with attaining better PCE values because of the better charge separation. In 2019, Arora et al. came up with a highly efficient perovskite/CuSCN/carbon C-PSC architecture (Figure 4c) delivering 18% PCE and retaining ≈95% of their initial efficiencies for >2000 h at the MPP under full-sun illumination at 60 °C (Figure 4d).^27^ Furthermore, Arora et al. demonstrated in their work that the use of CuSCN/carbon electrodes increased the shelf stability toward UV stress (Figure 4e) and by combining TiO_2_ with SnO_2_, the resistance toward UV light is further increased. More recently, Babu et al. demonstrated the use of carbon electrodes in large-area flexible PSCs (1 cm^2^ devices on polyethylene terephthalate (PET) foil) and achieved 15.8% PCE with excellent stability (1000 h MPP tracking at 85 °C).^70^

### Costs and Processability

**Figure 5:**
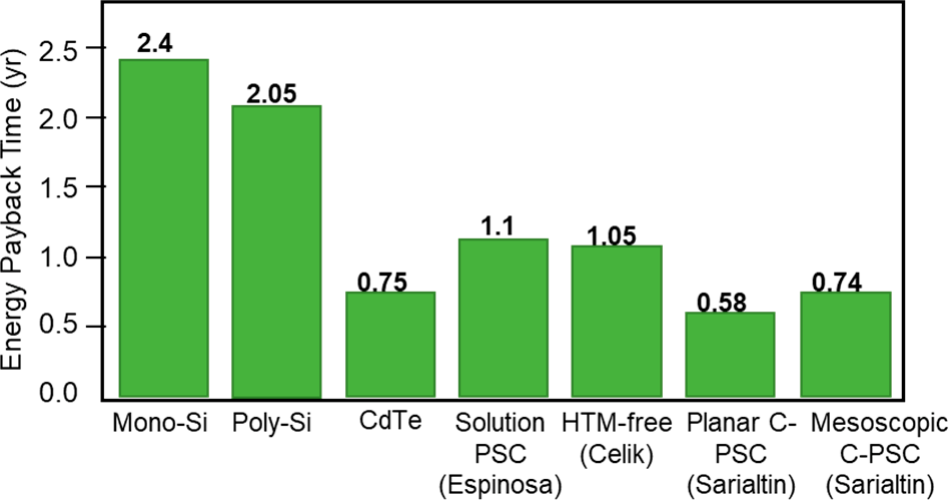
Energy payback time (EPBT) comparison of different C-PSC (HTM-free) architectures with the literature and first and second generation PV technologies. Planar PSCs with the carbon electrode show the lowest EPBT (0.58 yr) compared to other technologies. In particular, C-PSCs exhibit approximately 4 times lower EPBT than mono- and poly-silicon solar cells. Reproduced with permission from ref (73). Copyright 2020 AIP.

As mentioned, the use of carbon-based electrodes is a much cheaper alternative to noble metals such as gold and silver, not only due to the lower price of the raw material but also because such electrodes do not require energy-intensive deposition processes. Recent studies support this statement.^71−73^ In 2019, Sarialtin et al.^73^ compared the energy payback time (EPBT) of first- and second-generation PVs (mono- and poly-Si and CdTe) to regular, full-architecture solution processed PSCs and carbon-based HTM-free PSCs of different architecture (planar PSCs vs mesoscopic PSCs) (Figure 5). It appears that PSCs are more cost-effective than silicon PVs and can easily compete with thin film PVs. Silicon PVs exhibit EPBT of more than 2 years (2.4 years for mono-Si and 2.05 years for poly-Si respectively), whereas CdTe solar cells show below unity EPBT (0.75 years). Regular solution processed PSCs^71^ and carbon-based HTM-free PSCs from 2016^72^ show EPBT of around 1 year, and most recent C-PSCs demonstrate impressively low EPBT of 0.58 year for the planar architecture and 0.74 year for the mesoscopic one,^73^ rendering them attractive on an economic perspective and much more than the competing technologies.

**Figure 6:**
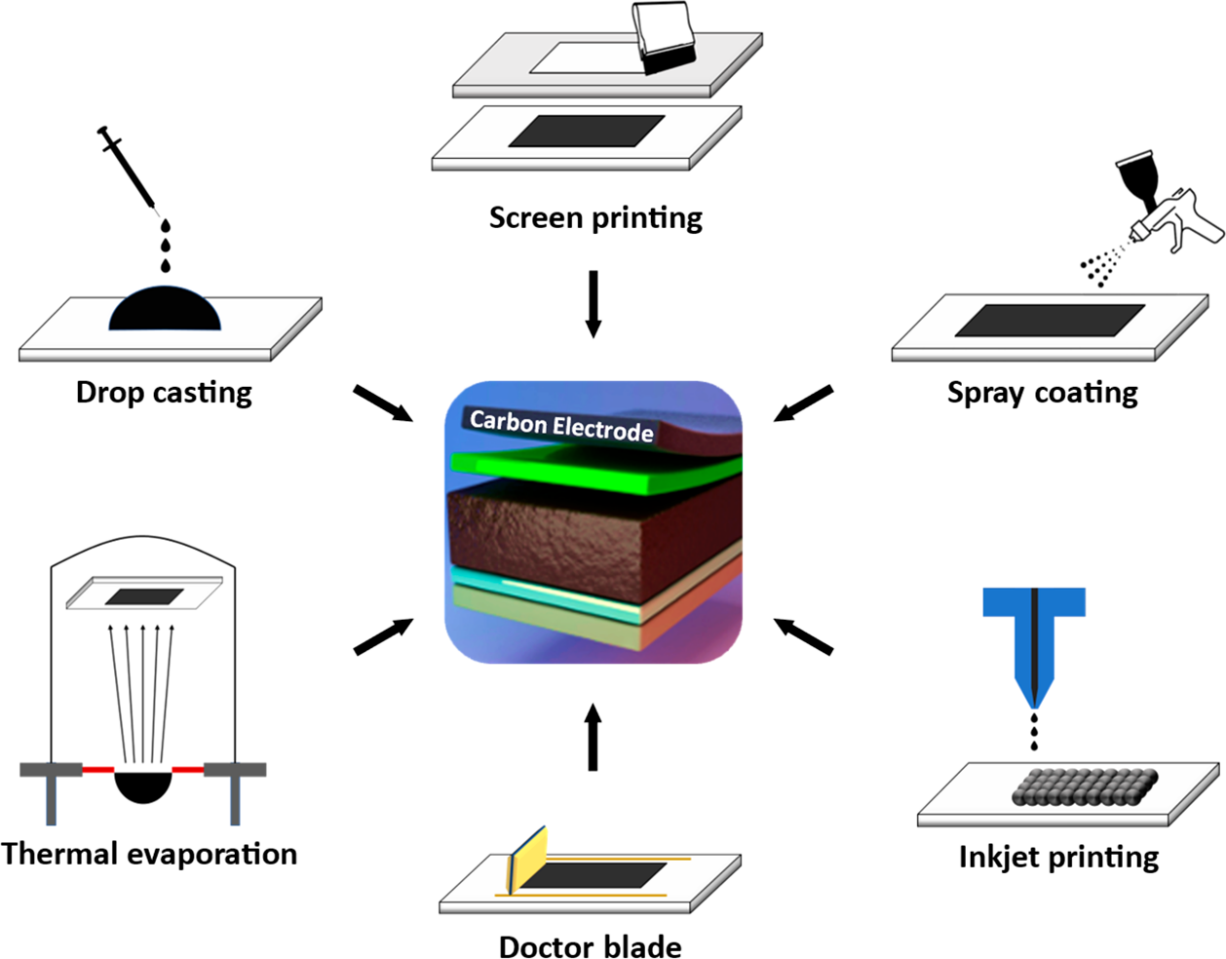
Schematic illustration of the different deposition methods available to use carbon electrodes in PSCs Adapted under the terms of the CC-BY 4.0 license (https://creativecommons.org/licenses/by/4.0/).^74^ Copyright 2021, The Authors, published by MDPI. Apart from thermal evaporation, which might be energy-intensive, all the other depicted methods represent simple deposition techniques and are, therefore, scalable procedures toward the industrialization of carbon-based PSCs. The ability to readily deposit carbon electrodes on top of the perovskite/HTM layer with no further treatment also constitutes a big step forward in the industrial development of C-PSCs.

Regarding processability, the transition from noble metals to carbon electrodes represents a big step toward the industrialization of PSCs. Effectively, noble metals require relatively energy-intensive deposition processes, whereas carbon electrodes can be effectively deposited by several different, simple, and scalable methods such as doctor blading, inkjet printing, drop-casting etc., as illustrated in Figure 6.^74^

## Flexible Device Design for Carbon Based-PSCs

**Figure 7:**
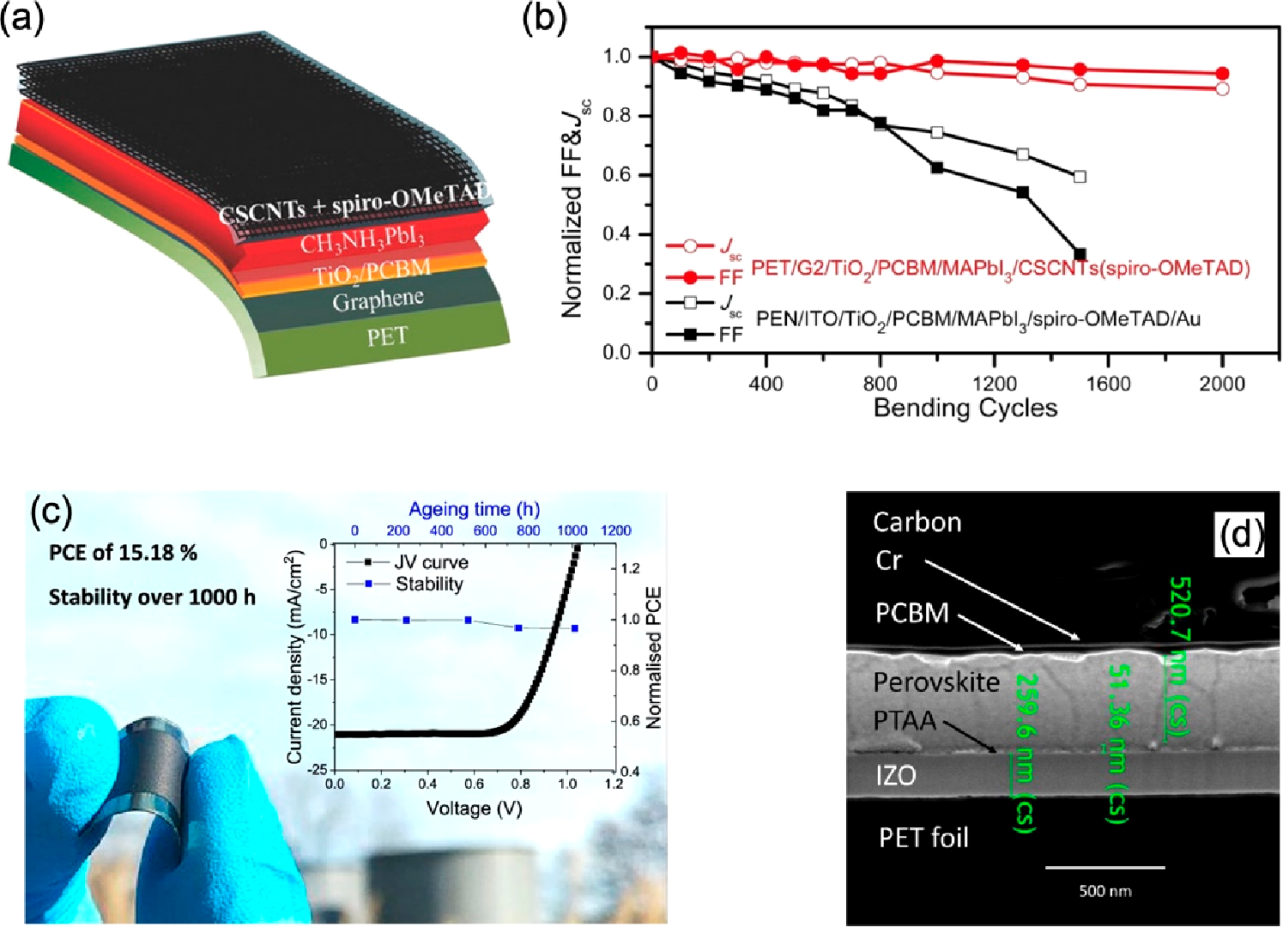
(a) Schematic and (b) *J*_sc_ and FF evolution of the ITO/PEN-based and all carbon-based FPSC versus bending cycles. (a, b) Reproduced with permission from ref (78). Copyright 2018 Wiley. (c) Optical image, *JV* curve, and stability corresponding to the Cr/C-PSC structure. (d) Cross-sectional SEM (scanning electron microscopy) image of a PSC with Cr as a buffer layer. (d, e) Reproduced with permission from ref (79). Copyright 2020 American Chemical Society.

Flexible solar cells (FSC) possess desirable attributes of lightweight, bendability, and mechanical durability, which are the ideal choice for portable wearable technology,^75^ integrated photovoltaic housing,^76^ aerospace, and various military fields.^77^ C-PSC is beneficial for a flexible device with its low-temperature preparation and large-area printing characteristics, and carbon electrodes with stable mechanical properties also enhance the environmental adaptability against bending. Luo et al. fabricated the all-carbon-based flexible perovskite solar cell with PET as the substrate, graphene as the transparent electrode, and cross-stacking carbon nanoparticles (CSCNPs) as the back electrode. The structure is shown in Figure 7a.^78^ After they optimized CSCNPs and the number of layers of graphene, the PCE of the champion device with Spiro-OMeTAD as the HTM reached 11.9%. In the bending test, the conversion efficiency of the reference group device, which is popularly used as the transparent electrode (ITO/PEN), dropped to 13% of its initial value after 1,500 bending cycles (Figure 7b). However, all carbon-based flexible PSC (C-FPSC) still maintained 84% of the initial conversion efficiency after 2,000 bending cycles, demonstrating bending endurance.^78^ Babu et al. fabricated large-area (1 cm^2^) high-efficiency C-FPSCs by processing carbon paste at low temperature (100 °C) and introducing an ultrathin chromium (Cr) buffer layer between the ETL and carbon electrodes, and the PCE reached 15.18% (Figure 7c).^79^ As an interlayer, Cr (Figure 7d) not only enhances the flexibility but also effectively facilitates electron transfer between PCBM and the back-contact carbon electrode, enabling the champion device to obtain the highest reported efficiency for flexible PSCs with carbon electrodes. From MPP and thermal (85 °C) aging tests, compared to the Cr/Ag electrode device which lost nearly 20% of its initial PCE after only 30 h, the carbon-based device still retained over 80% efficiency after 1000 h, demonstrating its remarkable thermal stability.^79^ Regarding flexible device design, the compatibility of carbon-based materials with FPSCs in the device design is greatly facilitated by roll-to-roll production methods, which is critical for the commercialization of wearable electronics.^80^

## Proposed n–i–p Architecture

**Figure 8:**
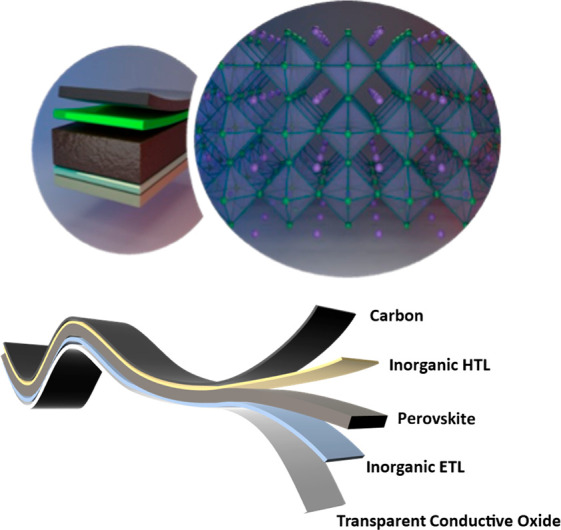
Proposed architectures for efficient, cost-effective, and stable perovskite solar cells. The perovskite is deposited on top of an inorganic electron conductor and covered by an inorganic hole conductor. Finally, the device is completed by depositing a carbon electrode on the inorganic hole conductor layer.

The most crucial bottlenecks impeding the commercialization of PSCs are cost and stability. Therefore, envisaging an overall architecture that would lead to a stable, cost-effective, and efficient device is mandatory for moving forward. In this section, the different layers of the PSC are revisited, and recommendations are made for reaching the champion device architecture, which is schematically represented in Figure 8.

### Electron Transporting Layer Optimization

The first layer in the regular architecture (n–i–p) of a PSC is the ETL. This layer is responsible for conducting the electrons and blocking the holes, so it must have a high carrier extraction rate and a low combination rate. TiO_2_ has been used as the typical n-type ETL material due to its high transmittance in the visible light region, low cost, chemical stability, nontoxicity and easy properties-tunability.^81^ Nevertheless, titania suffers from ultraviolet (UV) illumination instability due to photocatalytic activity, compromising the reproducibility and stability of the PSC over time.^82^ In fact, several reports have shown that the perovskite layer degrades under illumination due to photoelectron accumulation and trapping at the c-TiO_2_/perovskite interface.^83^ SnO_2_ has been more recently put forward as a good ETL candidate because of its higher conductivity (2 orders of magnitude higher), better optical transmittance, wider bandgap (3.6 vs 3.2 eV), and possible low temperature processing.^84^ Guo et al. showed that the partial incorporation of SnO_2_ nanoparticles in TiO_2_ precursor solution resulted in a TiO_2_/SnO_2_ nanocomposite which improved the cell efficiency because of the higher conductivity and the wider bandgap (3.9 eV).^82^ It also resulted in a faster charge extraction, reduced JV hysteresis, and improved long-term stability. Furthermore, Li et al. demonstrated that when the SnO_2_ ETL is replaced by phenyl-C61-butyric-acid-methyl-ester (PCBM, a benchmark organic ETL) in a 1 cm^2^ MAPbI_3_ perovskite module, the module production cost rockets to 801% of the initial costs and its LCOE is increased by 286%.^30^ Finally, Abuhelaiqa et al. demonstrated that a stacked bilayer of SnO_2_/TiO_2_ electron extraction film is a promising way to enhance the device stability without compromising the performance.^83^ The SnO_2_ was found to have a passivation effect, suppressing charge recombination with the perovskite layer and improving the optical durability. A long-term stable, efficient, and cost-effective perovskite device thus needs to have a layer of ETL that incorporates inorganic TiO_2_/SnO_2_ instead of organic benchmark materials.

### Perovskite Absorbing Layer Optimization

The amorphous or low crystallinity nature of the grain boundaries in polycrystalline perovskite (PC-PVK) thin films is responsible for the poor thermal stability, as it enhances detrimental ions migration, which in turn leads to the decomposition of the perovskite crystalline network and, thus, to the death of the PSC.^18b,85−88^

In recent years, single crystal (or monolithic) perovskite systems began to attract increasing attention from the PSC community with the development of new routes to prepare large area single crystal perovskite (SC-PVK).^89^ Effectively, SC-PVK presents many key advantages compared to their polycrystalline equivalents: being free of grain boundaries, SC-PVK offers better surface quality with orders of magnitude fewer defect density^90^ and extraordinarily improved optoelectronic properties such as much longer charge carrier diffusion length,^91,92^ reduced trap densities,^82^ extended absorption spectrum,^92,93^ and suppressed ion migration phenomenon.^94^ Due to the highly crystalline structure of the absorber, SC-PVK also exhibits drastically enhanced thermal stability, with a thermal decomposition temperature of up to 240 °C reported for SC perovskites compared to 150 °C reported for PC-PVK thin films.^95^ Furthermore, the highly pure crystalline nature of SC-PVK also makes them chemically more stable toward oxidation and hydrolysis, two of the main chemical degradation processes responsible for the degradation of PSCs when exposed to the natural atmosphere.

An impressive case of SC-PSC operational stability was demonstrated by Song et al.’s stand-free lateral structure devices, as no degradation at all was observed after 200 h of continuous operation at the MPP conditions, the devices still delivering 100% of their initial efficiency.^96^ Such operational stability without encapsulation is outstanding, likely unprecedented in the field of PSCs and represents one of the major arguments for focusing the research more toward single crystal PSC (SC-PSC) rather than polycrystalline PSC (PC-PSC). Effectively, SC-PSC does not compete with PC-PSC yet in terms of PCE, especially lateral structure SC-PSC, but the rapid progress demonstrates that SC-PSC has a very strong potential for competing with other PVs ultimately.

The PCE of SC-PSCs (or monolithic PSCs) shows an exceedingly fast increase. From an initial value of 1.73% in 2016,^97^ the PCE of SC-PSC already jumped to 21.09% in three years, which is already almost competing with their polycrystalline counterparts.^98^ Two vital factors drive the PCE of SC-PVK: the light-absorption depth and the carrier-diffusion length.^99^ The light-absorption depth determines the minimum thickness of the single crystal needed to harvest light efficiently, while the carrier-diffusion length defines the maximum thickness of the SC-PVK at which the photogenerated charge carriers can still be efficiently harvested at the selective contacts, reducing the overall recombination losses. The SC-PVK thus has to be thick enough to harvest light efficiently but thin enough to ensure that the photogenerated charges can reach the contacts to be collected. Besides PCE improvements, developing deposition methods suitable for industrial standards is another research direction required for the commercialization of SC-PSC. Indeed, the state of research regarding SC-PSC is still in its early stages, as SC-PSCs are a very recent technology.

Another point to consider with respect to the absorbing layer concerns its chemical composition.^100^ Most PSCs employ methylammonium (MA^+^) cations, which however suffer from a relatively wide bandgap and a decomposition to methylamine upon exposure to heat, light or moisture, impeding their large-scale production.^101^ In this regard, formamidinium (FA^+^) cations are currently considered an excellent alternative, as pristine FAPbI_3_ exhibits lower volatility, close to optimal Goldschmidt tolerance factor, and an absorption spectrum reaching the near-IR (840 nm), rendering FAPbI_3_ the most attractive perovskite layer for high-performing single-junction PSCs.^101,102^ Unfortunately, there is a phase transition from the black α-phase of thin FAPbI_3_ films to the yellow δ-phase (photoinactive) at a temperature of less than 150 °C. Many attempts have been made to stabilize the black phase by mixing FAPbI_3_ with MA^+^, Cs^+^, and/or Br^–^ ions, but this results in a blue-shift in the absorbance and phase segregation under operational conditions.^103^ Other promising stabilization approaches have been put forward recently: precursor engineering to fundamentally stabilize the pure phase; improving the stability of its internal structure (adjusting lattice strain using additives, passivators or transporting layers); and passivating the defects of the phase-pure α-FAPbI_3_.^103^

### Inorganic HTM

The critical role that inorganic HTM layers can play in yielding efficient, cost-effective, and stable PSCs has been extensively explained in this review (Figure 8). First, the HTM layer is vital for efficient hole extraction from perovskites. This is reflected by the higher *V*_OC_ obtained when using HTM, in contrast to HTM-free devices, which suffer from higher non-radiative charge-carrier recombination rates. Second, inorganic HTM is inexpensive and chemically and thermally stable, leading to an exemplary stability for PSCs. Moreover, they are easily processed using a large array of different deposition methods suitable to industrial standards. Particularly NiO_x_ and MoS_2_ have shown thus far the best results using spray coating, which can readily be used to deposit high-quality films of a large area.

### Carbon-Based Counter Electrode

The substitution of the noble metal with a carbon-based electrode is the second cornerstone of this review (Figure 8). This is due to the much cheaper cost of carbon, in addition to its excellent stability under harsh conditions, moisture resistance, superior electrical conductivity, flexibility, easily tuned properties, and processability via simple deposition methods. Moreover, it must be a defect-free interface with the HTM layer for efficient charge collection. A self-adhesive carbon electrode was demonstrated as a practical example.

## Conclusion

Perovskite solar cells are emerging as the most promising photovoltaic technology, showing the potential to supersede any other emerging PV technology in terms of efficiency, production costs, and EPBT. What renders PSCs ultimately attractive is that they exhibit much lower production costs than their silicon counterparts, regarding both the extraction of the raw materials and their transformation into photovoltaic-efficient devices. However, to envision a PSC architecture that is commercially viable and reachable to the market, a mind shift must be made. Instead of putting efficiency forward as the main objective despite poor stability, an averagely efficient PSC with high stability is more desirable. In fact, a lifetime of 15 years with an average PCE of 19% (with a module size of at least 100 cm^2^) was suggested as a threshold for real applications, which is still a long way from the current status. This review shows that incorporating a carbon layer as a back contact instead of noble metals and employing inorganic HTMs instead of organic ones are two cornerstones for achieving optimal stability. Other optimizations in the absorbing and electron-transporting layers are also suggested as additional factors for a stable single-junction architecture. More studies related to long-term stability are still needed, and the recommended architecture in this work offers one potential solution to the problem.
